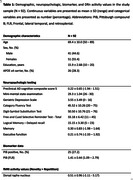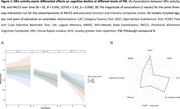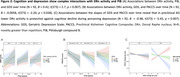# Lower dorsal raphe nucleus activity reflects resilience to Aß‐related cognitive decline in preclinical Alzheimer’s disease individuals with mild depressive symptoms

**DOI:** 10.1002/alz.095635

**Published:** 2025-01-09

**Authors:** Timothy Lawn, Prokopis C. Prokopiou, Lukas Heinrich, Donovan Nancy, Kathryn V Papp, Dorene M Rentz, Reisa A Sperling, Keith A Johnson, Heidi I.L. Jacobs

**Affiliations:** ^1^ Massachusetts General Hospital and Harvard Medical School, Boston, MA USA; ^2^ Gordon Center for Medical Imaging, Massachusetts General Hospital, Harvard Medical School, Boston, MA USA; ^3^ Athinoula A. Martinos Center for Biomedical Imaging, Massachussets General Hospital and Harvard Medical School, Boston, MA USA; ^4^ Brigham and Women’s Hospital and Harvard Medical School, Boston, MA USA; ^5^ Center for Alzheimer’s Research and Treatment, Department of Neurology, Brigham and Women’s Hospital, Harvard Medical School, Boston, MA USA; ^6^ Brigham and Women’s Hospital, Harvard Medical School, Boston, MA USA; ^7^ Center for Alzheimer’s Research and Treatment, Brigham and Women’s Hospital, Massachusetts General Hospital, Harvard Medical School, Boston, MA USA; ^8^ Athinoula A. Martinos Center, Massachusetts General Hospital, Boston, MA USA

## Abstract

**Background:**

The neuromodulatory subcortical systems are among the earliest brain regions to accrue pathology in Alzheimer’s disease (AD), contributing to cognitive and non‐cognitive symptoms. Monoaminergic nuclei, such as the dorsal raphe (DRn), modulate mood, cognition, and arousal. Their pathological perturbation is proposed to induce initial hyperexcitability followed by decreased activity, which may therefore be associated with the neuropsychiatric and cognitive symptoms of preclinical AD. Here, we associated novelty‐related activity of the DRn to longitudinal neuropsychiatric and cognitive changes in preclinical AD.

**Method:**

Ninety‐two older adults from the Harvard Aging Brain Study (mean age = 69.4±10, 51 females, **Table 1**) underwent baseline functional MRI where they were presented unfamiliar and familiar face‐name pairs organized within blocks of novelty and repetition to extract DRn novelty‐related activity. They additionally completed ^11^C PiB‐PET scanning to assess neocortical Aβ (cut‐off: 1.324 DVR, PVC) as well as annual neuropsychological testing (mean = 5.21±1.91 years). Robust linear regressions tested cross‐sectional associations, and robust linear mixed effects models assessed longitudinal associations, of DRn activity with cognitive decline or increases in depressive symptoms, in interactions with PiB (adjusted for age, sex, and years of education).

**Result:**

DRn and PACC5 were not associated at baseline nor in interaction with PiB (p<0.05). Longitudinally, lower DRn activity was associated with steeper cognitive decline at elevated PiB (**Figure 1A,** *B* = 0.092, *t*(374) = 2.65, *p* = 0.008). This was particularly driven by the category fluency and logical memory components of PACC5 **(Figure 1B)**. Conversely, higher DRn activity was associated with worsening of depressive symptomatology at high PiB (**Figure 2A**, *B* = 0.42, *t*(373) = 2.7, *p* = 0.007). To reconcile these opposing findings, we related both slopes: in preclinical AD with increasing depressive symptoms, individuals with higher DRn activity exhibited steeper cognitive decline, compared to those with lower DRn activity (N = 92, *B* = ‐0.48, *t*(81) = ‐5.43, *p* = 0.000).

**Conclusion:**

These findings indicate that lower DRn activity may reflect resilience to cognitive decline in preclinical AD individuals with worsening depressive symptoms. However, high DRn activity in those with worsening depressive symptomatology signifies cognitive deterioration. Future work including serial imaging is required to model the temporal evolution of serotonergic neuromodulation and its relation to disease progression.